# Combined Staged Surgery and Negative-Pressure Wound Therapy for Closure of a Giant Omphalocele

**DOI:** 10.1155/2021/5234862

**Published:** 2021-05-25

**Authors:** Vlad Laurentiu David, Mihai Cristian Neagu, Aurelia Sosoi, Maria Corina Stanciulescu, Florin George Horhat, Ramona Florina Stroescu, Calin Marius Popoiu, Eugen Sorin Boia

**Affiliations:** ^1^Department of Pediatric Surgery and Orthopedics, “Victor Babes” University of Medicine and Pharmacy, Timisoara, Romania; ^2^Department of Microbiology, “Victor Babes” University of Medicine and Pharmacy, Timisoara, Romania; ^3^Department of Pediatrics, “Victor Babes” University of Medicine and Pharmacy, Timisoara, Romania

## Abstract

The management of giant omphaloceles had always been a point of interest for the pediatric surgeons. Many surgical techniques were proposed, but none of them succeeded to become the standard procedure in closing the congenital abdominal defect. We present a case of giant omphalocele in which we used staged surgical closure combined with a prosthetic patch, with negative-pressure therapy and, finally, definitive surgical closure. Even though a major complication occurred during the treatment, we were able to close the defect without any prosthetic material left in place.

## 1. Introduction

Exomphalos (omphalocele) is a congenital defect located at the site of the umbilical cord insertion [1]. Omphalocele is considered a major form when the diameter of the defect is larger than 5 cm [1]. Usually, large defects contain most of the small bowel and/or more than 50% of the liver within the sac [1]. The management of large omphaloceles had always been a point of interest for the pediatric surgeons [2,3]. Even though primary surgical repair is the ideal treatment option, it is not possible in most cases of large defects. Due to lack of content, the abdominal cavity cannot be formed properly. Hence, there is not enough space to accommodate all the herniated organs. So, in this instance, other surgical options have to be considered: staged repair, silobag, prosthetic covers, or conservative management with delayed closure [1–3].

We present a case of giant omphalocele in which we were forced to use a sequence of treatment methods: staged closure using the Fufezan technique [4] followed by a Gore-Tex patch combined with negative-pressure therapy and, finally, definitive surgical closure.

## 2. Case Presentation

A male newborn was delivered by C-section at an estimated gestational age of 37 weeks with a birth weight of 2600 g. A large abdominal defect was diagnosed by ultrasound in the 19th week of gestation. The fetal ultrasound revealed a 3.5 cm/2.6 cm mass of bowel loops and the liver contained within a membrane in front of the abdominal wall.

**Figure 1:**
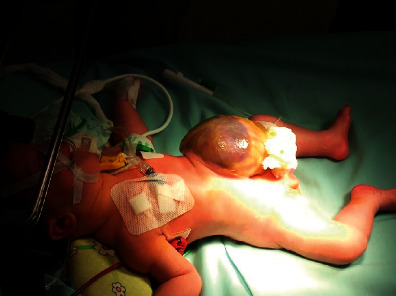
Giant omphalocele containing a significant portion of the liver.

At birth, examination of the abdomen revealed a large omphalocele (app. 8 cm in diameter) with an intact membrane containing part of the liver and small bowel loops (Figure 1). The echocardiogram showed a small ventricular septal defect with patent ductus arteriosus. G-band analysis showed no chromosomal abnormality and normal karyotype. No other congenital abnormalities were detected. Due to the size of the abdominal wall defect, we decided to perform staged closure of the omphalocele using partial resection of the omphalocele membrane (Fufezan technique [4]).

## 3. Surgical Procedure

**Figure 2:**
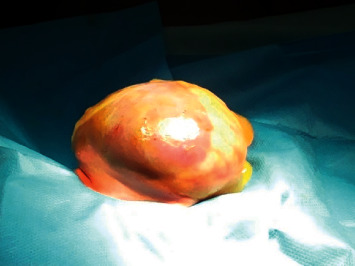
The omphalocele after the first stage of the procedure.

The procedure was performed 24 hours after birth. We performed an incision on approximately half of the circumference of the omphalocele membrane near the junction with the normal skin, at the lower pole of the defect. We detached the membrane from the underlying bowel loops and resected a slice of the membrane comprising approximately half of the membrane surface. The content of the sac was gently reduced into the abdominal cavity, and the remaining membrane was reattached using isolated nonabsorbable suture at the abdominal wall (Figure 2). The membrane was dressed with moisture sterile draping.

On the eight day of life, we performed the second stage of the procedure. In the same manner as in the first stage, we detached, partially resected, and reattached the membrane on to the abdominal wall further reducing the content of the omphalocele sack into the abdominal wall. The abdominal cavity was still not large enough to accommodate all the herniated organs.

On day 17 of life, we went into the operating theatre for the third time. We further reduced the herniated organs into the abdominal cavity till the point where all of the organs were inside the cavity. However, the defect could not be closed. We could no longer use the omphalocele membrane to further patch the defect which by now was dry and friable. So, we excised the remaining membrane, and then, we closed the defect using a Gore-Tex mesh. We further mobilized skin flaps and used them to cover the Gore-Tex mesh.

**Figure 3:**
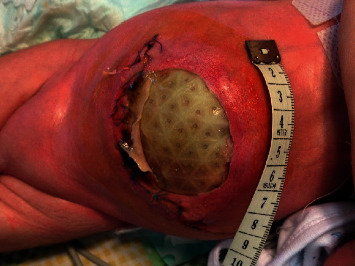
Skin necrosis with wound dehiscence and exposure of the Gore-Tex mesh.

Unfortunately, 5 days later (day 22 of life), the skin overlying the Gore-Tex mesh dehisced and a large portion of the Gore-Tex patch became exposed (Figure 3).

**Figure 4:**
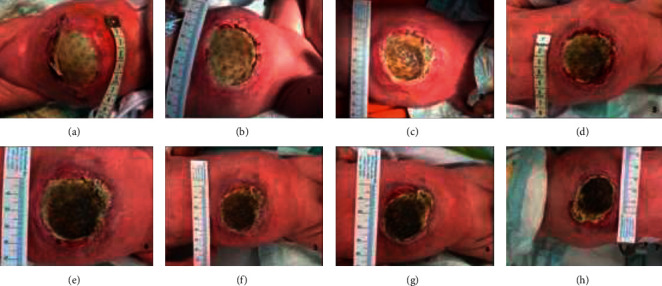
The abdominal defect through the NPWT. Initial aspect after the wound dehiscence (a) and after each of the 7 dressings (b–h).

**Figure 5:**
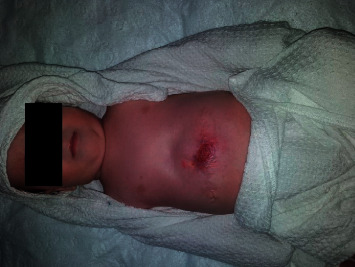
The wound at the time of discharge from the hospital. The abdominal wall was closed with only a small skin defect covered by granulation.

We decided to use a negative-pressure wound therapy (NPWT) combined with an antimicrobial silver dressing. The silver dressing was applied over the Gore-Tex patch and the negative-pressure system over it. We set the pressure at 40 mmHg, continuous negative pressure. The dressing was changed, and the wound was re-evaluated at 4 days of interval. The inflammation decreased, and the necrotic debrides were cleared after the first two dressings. Granulation formed at the edge of the tegument, and the wall defect became progressively smaller from approximately 7 cm to 3 cm diameter. At day 50 (28 days of NPWT), we were able to remove the Gore-Tex mesh and closed the wound (Figure 4). The patient was discharged 10 days later (Figure 5).

## 4. Discussion

The management of giant omphalocele is still debated among various methods of treatment, in using both surgical and nonsurgical approaches. The mainstay of treatment of such anterior abdominal wall defects is to reduce the herniated viscera into the abdomen and to close with the fascia and skin to create a solid abdominal wall. It is unanimously accepted that surgical closure of the defect should be the first choice and the conservative methods should be reserved for those cases where the patient is not able to sustain a major surgical procedure (associated malformations, shock, etc.) [1]. With regards to the surgical procedure, it is up to the surgeon to make the choice how to close the defect and in how many steps. However, with large defects and large visceral abdominal disproportion, primary closure should not even be attempted [5].

In our case, we preferred to use a staged closure of the defect. The child was well enough at birth to undergo the surgical procedure. We used the membrane omphalocele as a temporary cover of the herniated viscera as described by Fufezan et al. [4]. This method is similar with the use silobag temporary containers for the herniated viscera except that it uses the omphalocele membrane as a temporary cover. The method was validated decades ago in an era when silobags were not yet available [4]. We were able to progressively reduce the content of the sac into the abdominal cavity in 2 steps, and in the 3rd, we closed the remaining wall defect using a Gore-Tex patch, without inducing a compartment syndrome. The use of the Gore-Tex patches is a not a novelty in treating abdominal wall defects such as omphaloceles [1]. However, their use is not without risks. In our case, the skin-covering patch became necrotic and ruptured five days after surgery. This happened due to excess tension and lack of underlying vascular support.

Complications due to primary closure of giant omphaloceles are alarming [6,7]. In a survey conducted in 2011, a review by van Eijck et al. showed that the mean postoperative herniation rates were the highest in primary closure of large omphaloceles (58%) and the lowest in nonoperative delayed closure (9%) [3]. This means that an alternative strategy is necessary in GO cases with large tissue defects and large visceral abdominal disproportion, and in such cases, primary closure should not even be attempted [8–10]. Nowadays, most of the surgeons use temporary covers such as silobags, with good outcomes [8]. The use of conservative treatment that results in early skin cover by secondary wound healing is not without drawbacks. The re-epithelization time on the omphalocele sac can take 2-3 months, and the remaining eventration has to be closed later on [11].

The two main complications possible after closure of the congenital abdominal defects are compartment syndrome, due to excessive intra-abdominal pressure and wound dehiscence with visceral exposure [12,13]. Even though we were able to avoid abdominal hypertension by using stage repair and the Gore-Tex patch, rupture of the skin occurred after a few days after the definitive closure attempt. We were then forced to find a solution to cover the skin defect and protect the abdominal viscera from external exposure. The Gore-Tex patch was in place and was ensuring structural strength to the fascial sheet of the abdominal wall. So, in the absence of the compartment syndrome, we considered it is not wise to remove it. However, the Gore-Tex patch does not have insulation proprieties, so the abdominal cavity was not insulated from the external environment. The solution we found was to use a negative-pressure wound therapy system (NPWT) to temporarily close the skin defect. NPWTs are long used in the treatment of large or complicated skin wounds, infected wounds, etc. [14]. NPWT is seldom used also for congenital or acquired abdominal wall defects [15–18]. NPWT was applied directly over the omphalocele membrane to reduce the size of the defect and promote the reintegration of the herniated viscera [16]. In other instances, it was used for complicated omphalocele with a ruptured membrane and the NPWT dressing was applied directly over the exposed viscera [17]. In both instances, the NPWT was effective in cleaning the wound and promoting the defect reduction and viscera reintegration [16–18]. In our case, even though it took 28 days and 7 consecutive dressings, NPWT proved to be useful. There were several facts we had to consider when setting the NPWT system over the abdominal defect. First, we placed the vacuum dressing over the Gore-Tex patch, which acted as a protective barrier for the intra-abdominal organs. Direct suction pressure over the abdominal organs and contact with the silver dressing would have induced severe lesions. Previous studies' recommendations are to set the vacuum system over a protective mesh when use over and abdominal defect [15]. Secondly, we had to assess which is the optimum interval between dressing changes. Most of the dressings were changed in the operating theatre under sedation, so we prolonged the interval between dressings as much as possible. Last, since there are no clear recommendations, we set the pressure to the minimum of the device, −40 mmHg. We do not know if higher pressure would have a negative (or positive) impact, but since it was effective, we kept it as it is. The wound was cleaned from necrosis debridement, granulation tissue formed at the ages of the hound, the abdominal cavity was protected from external exposure, and the wound decreased in size till we were able to safely close the defect.

## 5. Conclusions

Delayed closure should be the preferred treatment method in giant omphaloceles. Various methods are available, and with this case presentation, we remind one of the old but useful techniques, the Fufezan technique. Negative-pressure wound therapy is effective in the treatment of congenital abdominal defects and should be considered as a temporary closure and to promote wound healing and shrinking of the abdominal wall defect.

## Data Availability

Data supporting the reported results are available upon request from the corresponding author.
